# Biomarkers in Pediatric Nephrology—From Bedside to Bench and Back Again

**DOI:** 10.3390/jcm11195919

**Published:** 2022-10-07

**Authors:** Kinga Musiał

**Affiliations:** Department of Pediatric Nephrology, Wrocław Medical University, Borowska 213, 50-556 Wrocław, Poland; kinga.musial@umw.edu.pl

The progress in biomarker research is characterized by the perpetual quest for parameters that fulfill the strict criteria of sensitivity, specificity, ease and speed in performance and cost-effectiveness. The expectations towards the range of functions the ideal index should deal with are increasing too. Lack of differentiation between biomarkers useful in the assessment of risk, prediction, prevention, diagnosis, progression or recovery raises the probability of functional overlap. Although unintentionally, such striving for perfection and versatility may lead to a dead end if there is no translation between sophisticated parameters and their use in clinical practice.

The example of acute kidney injury (AKI) clearly shows how difficult such implementation to the bedside can be. Despite years of research and subsequent recommendations, no internationally recognized definition of AKI has introduced any biomarker of damage so far [1]. Thus, only serum creatinine, estimated glomerular filtration rate, and urine output, condition clinical decisions regarding diagnosis, prognosis and treatment of AKI.

The problem seems even more serious in the pediatric field, where both physiological processes and pathological conditions largely depend on age and anthropometric data, extending presumed normal range values of biomarkers from prematures and neonates to adolescents [2]. Moreover, the profit advantage over the risk is always one of the priorities in the pediatric population, so noninvasive methods would overrate the invasive ones. Therefore, the evaluation of biomarkers in the urine would prioritize their assessment in the blood and the use of micro-methods would gain more attention than assays requiring excessive blood sampling [3,4]. The progress is also evident in bringing personalized medicine to everyday practice, where individual approach and adjustment of biomarkers to specific patients is the rule [5].

On the other hand, the assessment of biomarkers in specific panels seems superior to the evaluation of single parameters [4]. Last, but not least, the demands towards assessed parameters increase with time. Thus, today diagnostic indices are just the prelude, whereas prognostic markers or predictive models based on big data and built up with the tools of artificial intelligence are highly expected [6].

Pediatric nephrology seems to take on this challenge, tailoring parameters to various specific demands, including those of the neonatal period, rare diseases, inherited disorders or peritoneal dialysis [7,8,9,10].

However, with their specificity and multiplicity of topics, kidney diseases of childhood and adolescence don’t make the search for ideal biomarkers easy. On the whole, understanding pediatric kidney meanders requires screening through the plethora of genetically conditioned, age-related, disease-specific and renal tissue-originating potential biomarkers. The pathology they illustrate is either restricted to clearly defined cells/milieu within the kidney, or to intrarenal interactions, or it triggers systemic engagement through interorgan crosslinks. Changes limited to the kidney are characteristic for the early phase of any renal pathology, then the systemic influence becomes the rule. However, it is usually challenging, because the dilemma whether the kidney is the culprit or the victim can remain unresolved. Not unexpectedly, the kidney may also play both roles at the same time, as it occurs in the case of proteinuria in the course of chronic kidney disease.

Meanwhile, the examples of cardiorenal and hepatorenal syndromes unravel the truth of complex links between hemodynamic, metabolic, oxidative, inflammatory and neurohormonal conditions originating from distant organs [11,12]. They also highlight the role of holistic approaches as the optimal way of diagnosing and treating the patient as a whole, not just the disease itself. Additionally, functional links between various organs have created the possibility of universalization among markers, e.g., those reserved for cardiovascular comorbidities revealed their usefulness in chronic kidney disease [13].

The most recent example of a systemic approach towards the disease is the COVID-19-related pulmonary-renal crosstalk, placing the kidney as the second most frequently affected organ during SARS-CoV-2 infection. The existence of the lung-kidney axis has underlined the complexity and dynamism of interactions between the two organs and has given stimulus to new discoveries in the area of biomarkers predicting the development of complications and the patients’ outcome [14,15].

In order to highlight the pathological background of a newly discovered disease, the molecular mechanisms of SARS-CoV-2 toxicity were analyzed within the clinical context. The first reports on the increased chemokine concentration, complement and coagulation overactivity stimulated a search for the molecular background of symptoms such as acute respiratory distress syndrome, thrombotic events or acute kidney injury [16,17]. Tracing the development of COVID-19 infection throughout various virus mutations enabled the acquisition of knowledge of SARS-CoV-2 structure, receptors, mechanisms of cell invasion and the way of systemic expansion with multiorgan affectation [14]. The invasion of SARS-CoV-2 was due to its entrance receptor, ACE-2, spreading all over the body and it was particularly abundant within the renal proximal tubules [18]. However, the direct viral toxicity was not sufficient and only its multiplication by overlapping innate immunity mechanisms, relied to the cascade activation of inflammation, necrosis, complement system, endothelial damage and thrombosis, could aggravate destructive mechanisms and lead to multiorgan failure [19]. The auto-amplification loops of several mechanisms, together with the interactions of circulating immune cells, gave way to the efficacious spread of stimulants such as chemokines or products released during cell damage [20]. Therefore, the subsequent stages of COVID-19-related research allowed for the definition of all clinical phenomena and connections between them, then the testing of the hypotheses explaining the molecular background of observed pathologies, and finally the introduction of innovative therapies based on recently acknowledged facts [21,22].

Thus, the turnover of candidate biomarkers, as well as their diagnostic/prognostic/therapeutic potentials during COVID-19 infection, were analyzed from bedside to bench and back again, and also in children [15]. Such attitudes should be implemented into pediatric biomarker research routinely.

The special issue entitled “Pediatric and adolescent nephrology facing the future: diagnostic advances and prognostic biomarkers in everyday practice” contains articles written in the era when COVID-19 had not yet been a major clinical problem in children. Now that we know its multifaceted clinical course, complications concerning the kidneys, and childhood-specific post-COVID pediatric inflammatory multisystem syndrome (PIMS), the value of diagnostic and prognostic biomarkers in the pediatric area should be appreciated, and their importance ought to increase [23].

The readers will have the opportunity to get acquainted with the spectrum of all major aspects of biomarker use in pediatric nephrology: from neonatal to adolescent perspectives, from rare genetic disorders to civilization diseases such as obesity, diabetes, and hypertension, from transient acute kidney injury to fibrosis and irreversible end-stage kidney disease, as well as from in situ sources to systemic manifestations.

The content should convince both the scientists and clinicians that re-shaping the attitude towards biomarkers is the only way to build up a de novo strategy, first based on in-depth analysis of the changeable clinical picture, then bridging clinical to molecular data, and coming back to the patient with molecular solutions to clinical challenges. No matter how far we reach, the patient remains in focus and gives the final answer to the question of the potential usefulness of tested biomarkers. Therefore, let these articles give us courage to come back to the bedside and start using biomarkers in everyday practice for the patients’ good.

## Data Availability

Not applicable.
